# Delivery of IL-2 to the T Cell Surface Through Phosphatidylserine Permits Robust Expansion of CD8 T Cells

**DOI:** 10.3389/fimmu.2021.755995

**Published:** 2021-11-04

**Authors:** Alana MacDonald, Brandon Lam, John Lin, Louise Ferrall, Yu Jui Kung, Ya Chea Tsai, T.-C. Wu, Chien-Fu Hung

**Affiliations:** ^1^ Graduate Program in Immunology, Johns Hopkins University School of Medicine, Baltimore, MD, United States; ^2^ Department of Pathology, Johns Hopkins University School of Medicine, Baltimore, MD, United States; ^3^ Department of Oncology, Johns Hopkins University School of Medicine, Baltimore, MD, United States

**Keywords:** phosphatidylserine (PS), annexin V, interleukin 2 (IL 2), CD8 T cells, activation

## Abstract

The phospholipid phosphatidylserine (PS) is naturally maintained on the cytoplasmic side of the plasma membrane. Independent of apoptosis, PS is redistributed to the surface of CD8 T cells in response to TCR-mediated activation. Annexin V (AnnV) is a protein known to bind PS with high affinity and has been effectively utilized to anchor antigen to the surface of CD8 T cells. To expand these studies, we aimed to exploit TCR activation driven PS exposure as a target to deliver cytokine, namely interleukin-2 (IL-2), to the surface of CD8 T cells. This was accomplished using a novel chimeric fusion protein of annexin V and interleukin 2 (AnnV-IL2). *In vitro* analysis revealed that AnnV-IL2 is able to specifically bind PS on the T cell surface following TCR stimulation. Consequently, AnnV-IL2 proved to be significantly more effective at enhancing T cell activation compared to recombinant IL-2. *In vivo*, AnnV-IL2 promotes robust expansion of antigen-specific cells capable of interferon gamma (IFNγ) production when administered following peptide vaccination. Importantly, upon antigen rechallenge, AnnV-IL2 treatment mice demonstrated a stronger secondary expansion, indicating durability of AnnV-IL2 mediated responses. Our data supports the use of AnnV-IL2 to modulate antigen-specific T cell immunity and demonstrates that the PS-AnnV axis is a feasible mechanism to target diverse cargo to CD8 T cells.

## Introduction

The adaptive immune system is critical for defense against pathogens and providing long-term immunological protection. CD8 and CD4 T cells are an important arm of the adaptive immune system and the T cell repertoire is incredibly diverse with the potential for 10^7^-10^8^ unique T cell receptors poised to respond to a pathogenic challenge (1). Physiologically, CD8 T cells function to recognize and remove infected and malignant cells through antigen receptor mediated recognition of peptides presented on MHC class I molecules (pMHC). T cell priming against foreign antigens is a complex process requiring the integration of signal 1, through pMHC, and signal 2, through CD28 co-stimulation (2). The local cytokine milieu present during T cell priming is commonly referred to as “signal 3” and helps skew CD4 and CD8 T cells to the appropriate effector cell phenotype to achieve full T cell activation (2). IL-2 is a 15.5 kDa cytokine primarily produced by T cells as well as dendritic cells early in T cell activation and acts locally through autocrine or paracrine binding to the IL-2 receptor (IL-2R) (3). IL-2 signaling activates several key pathways including phosphorylated signal transducer and activator of transcription 5 (pSTAT5), mitogen-activated protein kinase (MapK)/extracellular signal-related kinase (Erk), and phosphoinositol-3-kinase (PI3K)/Akt pathways to promote survival, proliferation and differentiation of activated T cells (4). Moreover, it is well understood that the strength of IL-2 signaling influences cell fate through activation of different transcriptional programs. High concentrations of IL-2 present during antigen receptor activation is associated with the development of a short-lived, terminal effector cell phenotype, while in contrast, low concentrations of IL-2 are associated with the formation of long-lived memory cells (4).

The eukaryotic plasma membrane is carefully regulated to maintain an asymmetric distribution of phospholipids under homeostatic conditions. The anionic phospholipids, phosphatidylserine (PS) and phosphatidylethanolamine, are maintained on the inner leaflet, while phosphatidylcholine and sphingomyelin primarily compose the outer leaflet (5). Membrane asymmetry is compromised during programmed cell death, leading to re-localization of PS to the surface of cells (6). Phagocytic cells express a variety of PS scavenging receptors that facilitate apoptotic cell recognition and clearance (7–10). AnnV, a member of the annexin superfamily, is known to interact with phosphatidylserine with high affinity (K_D_ = 10^-9^ M), and for this reason, AnnV has been extensively developed for detection of apoptotic cells *in vitro* and *in vivo* (11–15). Additionally, the unique molecular structure of AnnV allows for the ability to self-organize into 2D arrays on the cell surface (16).

Our group and others have determined that PS exposure is not an exclusive event to apoptotic cell death (17–20). Following T cell activation, there is a transient redistribution of phospholipids and PS can be detected on the surface for up to 72 hours before membrane homeostasis is returned. Mao et el. designed a novel chimeric fusion protein that takes advantage of T cell derived PS to generate antigen-specific immunity through AnnV-mediated TCR cross-linking on CD8 T cells (17). The pMHC domain prompts signaling through the cognate TCR and triggers PS exposure, allowing the AnnV domain to engage PS and anchor the protein to the surface of the cell. The unique ability of AnnV to self-assemble will result in recruitment of additional AnnV-pMHC molecules and subsequent cross-linking of the TCR to drive a feed-forward loop of antigen-specific T cell activation. However, this method requires knowledge of a specific pMHC that activates a desired population of T cells and poses a translational limitation when antigen specificity is unknown.

In the present study we describe a novel, AnnV-based chimeric fusion protein designed to respond to plasma membrane cues associated with T cell activation. Our design exploits the high affinity PS-AnnV interaction to selectively deliver a cytokine with immunostimulatory properties, IL-2, to the surface of PS+ T cells to support activation without dependency on a specific pMHC. We aimed to address whether AnnV-IL2 has biological functionality and if targeted delivery of IL-2 would aid T cell activation. We demonstrated the ability of AnnV-IL2 to significantly increase the magnitude of the primary immune response and augment memory cell formation in comparison to IL-2. Fusion of IL-2 to the PS-binding protein, AnnV, proved to be a viable strategy to increase the population of effector CD8 T cells.

## Results

### T Cells Externalize Phosphatidylserine in a Non-Apoptotic Manner

**Figure 1:**
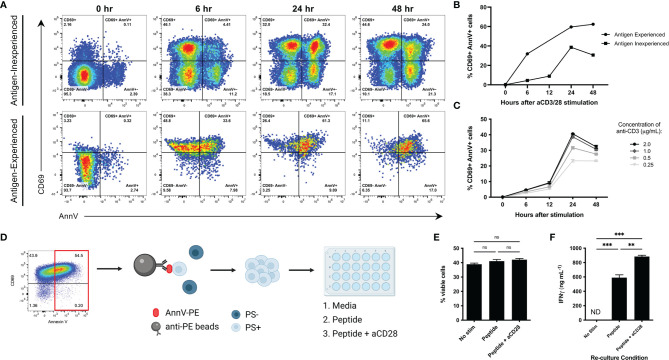
Characterization of transient externalization of phosphatidylserine in activated T cells. **(A, B)** Antigen inexperienced or antigen experienced CD8+ cells were cultured with anti-CD3 and anti- CD28 (antigen inexperienced cells only) and stained with CD69 and AnnV at the indicated time points. Live and activated CD8+ cells were gated on forward scatter and side scatter profiles. Representative dot plots from one sample are shown. **(C)** Frequency of CD69+ AnnV+ cells at the indicated time points for antigen inexperienced CD8+ T cells stimulated with the indicated concentrations of anti-CD3. **(D)** Schematic of experiment workflow. **(E)** Frequency of viable cells after 3 days of re-culture was quantified by flow cytometry. **(F)** ELISA for IFNγ in culture supernatant. Data is represented by mean ± SEM. P values were calculated by ordinary one-way ANOVA with the Tukey-Kramer multiple comparison test, and P < 0.05 is considered statistically significant. ** = < 0.01,*** = < 0.001. AnnV, Annexin V. NS, not significant. Schematic in 1D was generated using BioRender.com.

Several groups have reported changes in phospholipid distribution in T lymphocytes following T cell receptor (TCR) stimulation (17–19). Importantly, surface PS was not observed following exposure to proinflammatory cytokines such as interferon gamma (IFNγ) and IL-2, emphasizing that this phenomenon is antigen mediated (18). We first wanted to confirm whether CD8 T cells display surface PS following antibody-mediated activation. We utilized naïve CD8 T cells isolated from murine spleens and lymph nodes (antigen inexperienced) and *in vitro* generated memory cells (antigen experienced) to understand how both cell types respond to TCR stimulation. Indeed, both antigen inexperienced and experienced cells upregulated CD69, a marker of early T cell activation, and AnnV staining indicated that PS was externalized following TCR crosslinking (**Figure 1A**). We observed peak surface PS after 24 hours in antigen inexperienced cells and peak surface PS was maintained for 48 hours in antigen experienced cells (**Figures 1A, B**). To understand how the strength of T cell receptor (TCR) signaling impacts PS externalization, we cultured antigen inexperienced cells with increasing concentrations of anti-CD3 and measured PS exposure at the indicated time points. Increasing the level of T cell receptor stimulation resulted in a higher frequency of CD69+ AnnV+ cells after 24 hours (**Figure 1C**). Our finding that both antigen inexperienced and antigen experienced CD8 T cells briefly display PS on the surface is consistent with published literature.

Externalized PS is a signature event of apoptosis and serves as a key signal that facilitates uptake and clearance by phagocytic cells (7). We next wanted to confirm that PS exposure following T cell activation was not indicative of apoptosis and PS+ T cells were viable and functional. To accomplish this, CD8 cells were activated by anti-CD3 crosslinking to induce a high level of surface PS. PS+ cells were positively selected and re-cultured to the indicated conditions. The viability and cytokine production of the PS+ T cells was analyzed after 3 days (**Figure 1D**). PS+ cells cultured with peptide or peptide plus anti-CD28 were viable as determined by negative staining using a dead cell discrimination dye and light scattering characteristics (**Figure 1E**). In addition, they were able to produce high levels of IFNγ (**Figure 1F**). PS+ cells cultured in media alone remained viable, but unable to produce cytokine when the activating signals were removed (**Figures 1E, F**). This data confirms that surface PS following T cell receptor stimulation is not limited to cell death.

### AnnV-IL2 Engages Externalized PS and the IL-2R

**Figure 2:**
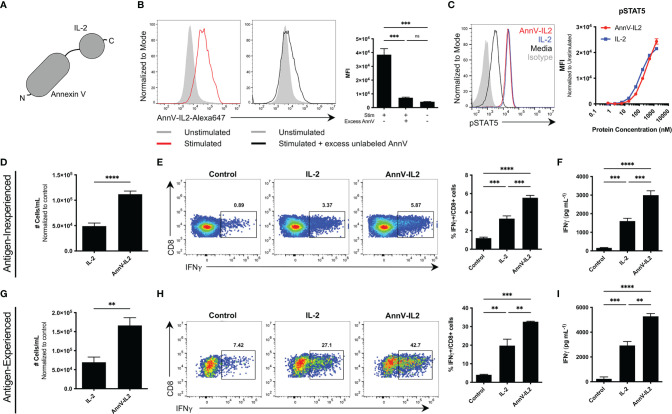
Comparison of the function of AnnV-IL2 and IL-2 *in vitro*. **(A)** Schematic of AnnV-IL2 construct. **(B)** Antigen experienced OT-1 cells were stimulated with anti-CD3 and anti-CD28 antibodies or left unstimulated for 5 hours. Cells were then pre-treated with or without excess unlabeled AnnV prepared in annexin V binding buffer for 30 minutes. Cells were washed and then stained with Alexa-647 labeled AnnV-IL2 or IL-2 protein. Histograms represent one sample from each group. **(C)** CTLL2 cells were IL-2 starved for 5 hours and then stimulated with the indicated concentration of AnnV-IL2 or IL-2 for 10 minutes and then immediately fixed. Cells were permeabilized and stained intracellularly with anti-pSTAT5 or isotype antibodies. **(D)** Antigen inexperienced or **(G)** antigen experienced CD8+ cells were cultured with anti-CD3 and anti-CD28 antibodies for 24 hours. After 24 hours, media was removed and replaced with media containing anti-CD28 with or without equimolar amounts of AnnV-IL2 or IL-2 and incubated for 5-6 days. Absolute cell counts were determined using a flow cytometer. **(E)** Antigen inexperienced or **(H)** antigen experienced CD8+ T cells were cultured with anti-CD3 and anti-CD28 in the presence of equimolar amounts of AnnV-IL2 or IL-2 for 3 days. Brefeldin A was added to culture media on day 3 and IFNγ production was quantified by intracellular flow cytometry. ELISA for IFNγ in supernatant of **(F)** antigen inexperienced or **(I)** antigen experienced cell cultures. **(D–I)** Control refers to cells stimulated with only anti-CD3 and anti-CD28. Data is represented by mean ± SEM. P values were calculated by ordinary one-way ANOVA with the Tukey-Kramer multiple comparison test, and P < 0.05 is considered statistically significant. ** = < 0.01,*** = < 0.001, **** = < 0.0001. AnnV, Annexin V; MFI, Median fluorescent intensity. NS, not significant.

To harness the targeting ability of AnnV and stimulatory property of IL-2, we generated a recombinant chimeric fusion protein by combining the respective DNA sequences. The AnnV domain is located at the N terminus and IL-2 domain is located at the C terminus (**Figure 2A** and **Supplementary Figure 1A**). We first wanted to test whether the AnnV domain of our chimeric construct maintained its ability to interact with exposed PS on activated T cells. To assess the PS binding capability of AnnV-IL2 by flow cytometry, CD8 T cells were activated and then stained with a fluorescently labeled AnnV-IL2 protein. We observed a shift in intensity with the stimulated cells versus the unstimulated cells, indicating that our protein bound to the cell surface (**Figure 2B**). To further investigate whether AnnV-IL2 was binding specifically to exposed PS, we pretreated activated cells with excess unlabeled AnnV to saturate PS binding sites prior to staining with AnnV-IL2. Cells pretreated with unlabeled AnnV had significantly reduced median fluorescent intensity (MFI), suggesting that AnnV-IL2 was able to target PS on the T cell surface (**Figure 2B**). Relatedly, we evaluated whether the IL-2 domain of the chimeric construct was biologically active and able to induce sufficient pSTAT5 downstream of the IL-2R. CTLL2 cells are dependent on IL-2 for growth and survival and are commonly used to assay IL-2 signaling events. Since CTLL2 cells require IL-2 supplemented media, we rested cells in media free of IL-2 for five hours to reduce pSTAT5 expression prior to being stimulated with AnnV-IL2 or IL-2. Phospho-flow cytometry analysis showed that AnnV-IL2 was able to induce comparable pSTAT5 expression as IL-2 and suggests that the fusion does not impede the ability to bind the IL-2R (**Figure 2C**). Together these data demonstrate that AnnV-IL2 is functional and able to interact and engage surface PS and the IL-2R.

### AnnV-IL2 Augments CD8 T Cell Proliferation and Effector Function *In Vitro*

The PS-AnnV axis has been successfully utilized to orchestrate T cell signaling events at the molecular level (17). In that regard, we hypothesized that AnnV-IL2 would augment CD8 T cell activation because AnnV would position IL-2 at the T cell surface. Antigen inexperienced or antigen experienced CD8 T cells were activated by anti-CD3/anti-CD28 and cultured in media supplemented with AnnV-IL2 or IL-2 protein. In both cell types, AnnV-IL2 resulted in greater counts of CD8 T cells (**Figures 2D, G**). Furthermore, AnnV-IL2 treatment led to a significantly higher population of IFNγ producing cells compared to IL-2 or media alone (**Figures 2E, H**). This observation was confirmed using an ELISA to measure IFNγ secretion in culture supernatant. As expected, AnnV-IL2 treatment resulted in higher levels of IFNγ in the supernatant of antigen-inexperienced and antigen-experienced CD8 T cells (**Figures 2F, I**). Since we were able to induce stronger activation independent of pMHC, we wanted to evaluate whether AnnV-IL2 could enhance activation following antigen-dependent TCR stimulation. We stimulated antigen-experienced OT-1 cells with cognate peptide in the presence of AnnV-IL2 or IL-2. Similar to our results with non-specific stimulation, we observed greater frequency of IFNγ-producing OT-1 cells with AnnV-IL2 (**Supplementary Figures 1B, C**). AnnV-IL2 was able to induce robust OT-1 activation at sub-threshold concentrations in the picogram range whereas peptide alone was unable to effectively activate OT-1 cells (**Supplementary Figure 1D**). Furthermore, AnnV-IL2 was more than 100-fold more potent than IL-2 at inducing IFNγ at low levels of TCR stimulation, highlighting the benefit of tethering IL-2 to the T cell surface (**Supplementary Figure 1D**). These results suggest that delivery of IL-2 to CD8 T cells through the AnnV-PS axis enables the generation of a larger population of IFNγ producing T cells following TCR activation.

In addition to CD8 T cells, natural killer (NK) cells express IL-2Rβγ and become activated *in vitro* in response to IL-2 (21). To better understand the effects of AnnV-IL2 on NK cell activation, we cultured murine splenocytes in the presence of AnnV-IL2 or IL-2. Both AnnV-IL2 and IL-2 induced robust NK cell expansion after 3 days (**Supplementary Figure 1E**). Interestingly, AnnV-IL2 induced a stronger cytotoxic response at the effective dose concentrations (**Supplementary Figure 1F**). Protein concentrations below 1μm did not incite an NK cell response and are likely sub-threshold, and high protein concentrations resulted in comparable IFNγ production in NK cells. This data indicates that AnnV-IL2 is able to expand NK cells *in vitro*.

### AnnV-IL2 Accumulates in the Spleen of Mice

**Figure 3:**
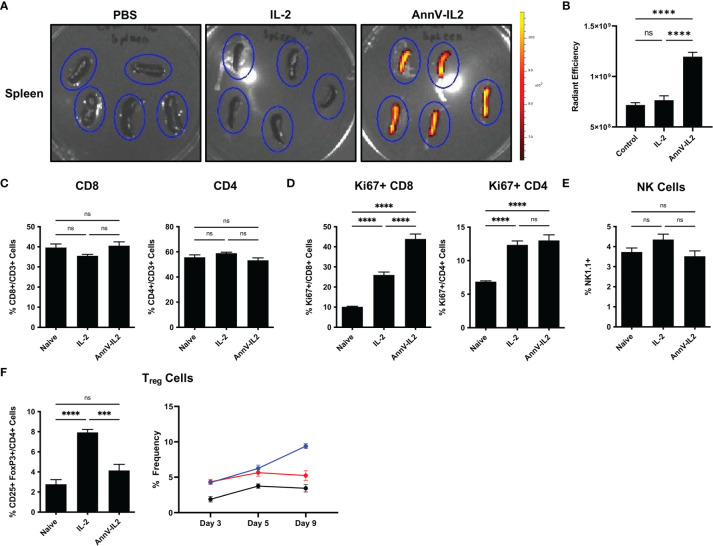
AnnV-IL2 accumulates in the spleen and induces Ki-67 expression in CD8 T cells. **(A)** Equimolar amounts of Alexa-647 labeled AnnV-IL2 or IL-2 protein was injected intravenously. After 4 hours the spleens were removed and imaged via IVIS imaging. Spleens are outlined in blue ovals, N=5 mice per group. **(B)** Quantification of fluorescence signal in the spleen **(C–F)** Mice were intravenously injected with AnnV-IL2, IL-2, or PBS (naive) once daily for 9 days total. PBMC’s were collected on day 9 and analyzed by flow cytometry. N=5 mice per group. Data is represented by mean ± SEM. P values were calculated by ordinary one-way ANOVA with the Tukey-Kramer multiple comparison test, and P < 0.05 is considered statistically significant. *** = < 0.001, **** = < 0.0001. NS, not significant.

IL-2 has a short circulating half-life of approximately 7 minutes and is rapidly degraded *in vivo* (22, 23). Since AnnV-IL2 has an additional protein domain and therefore a larger molecular weight relative to IL-2, we sought to understand how these modifications would affect the biodistribution profile of AnnV-IL2. Mice were intravenously injected with an equimolar ratio of fluorescently labeled AnnV-IL2 or IL-2 protein. After 4 hours, the spleen, lymph nodes, liver and kidneys were removed from the mice and *in vivo* fluorescence imaging indicated that AnnV-IL2 protein accumulated and was retained in the spleen of mice, whereas little signal was detected for IL-2 (**Figures 3A, B**). Due to the poor pharmacokinetic profile, IL-2 was likely cleared by the liver and kidney prior to imaging (**Figures 3A, B** and **Supplementary Figures 2A, B**). Interestingly, the lymph nodes did not retain either AnnV-IL2 or IL-2 protein after 4 hours (data not shown). This data indicates that AnnV-IL2 is retained longer in an immunologically relevant tissue and is positioned in closer proximity to IL-2R+ cells.

### AnnV-IL2 Preferentially Expands Activated T Cells *In Vivo*

IL-2 has been a cornerstone of the immunologic toolkit following its discovery as a T cell growth factor in the late 1970’s (24). Early *in vivo* data revealed that IL-2 could potently stimulate host immunity and has since been extensively investigated as an immunologic adjuvant for the treatment of disease (25–27). Therefore, we wanted to understand how *in vivo* delivery of AnnV-IL2 affects the immune profile with respect to IL-2. Mice were treated once a day for a total of nine days and peripheral blood was collected to monitor changes in global immune profile. We observed no significant differences in the frequencies of CD8 or CD4 T cells (**Figure 3C**). However, AnnV-IL2 induced a significantly higher frequency of Ki67+ CD8 T cells, indicating that cells are actively in the cell cycle and are likely undergoing proliferation (**Figure 3D**) (28). In CD4 T cells, AnnV-IL2 and IL-2 induced a comparable level of Ki67 expression (**Figure 3D**). In addition to conventional CD4 and CD8 T cells, IL-2 is an important cytokine for NK cells and CD4 T regulatory cells (Tregs) (3). To our surprise, we did not observe significant expansion of NK cells following *in vivo* administration of AnnV-IL2 or IL-2 (**Figure 3E**). We observed an increase in CD25+ FoxP3+ Tregs by day 5 in both AnnV-IL2 and IL-2 treated groups compared to naïve mice. By day 9, Treg levels remained elevated in IL-2 treated mice, while Treg populations in AnnV-IL2 treated mice were comparable with that of naïve mice (**Figure 3F**).

**Figure 4:**
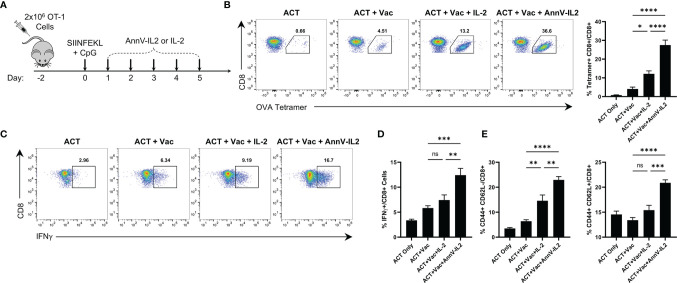
AnnV-IL2 expands adoptively transferred cells *in vivo*. **(A)** Schematic of experimental design. Briefly, naive C57BL/6 mice received 2x106 OT-1 cells intravenously on day -2, followed by intraperitoneal injection of the peptide corresponding to the immunodominant epitope of OVA (OVA257-264, SIINFEKL) and TLR9 agonist, CpG, on day 0. Mice were treated with equimolar amounts of AnnV-IL2 or IL-2 intraperitoneally once daily for a total of 5 days. On day 8, peripheral blood mononuclear cells were collected by submandibular bleeding. Dots plots are representative of one mouse in each group. N=5 mice per group. **(B)** Antigen-specific cells were quantified by tetramer staining. Bar chart depicts frequency of OVA257-264-specifc CD8+ T cells **(C)** PBMC’s were stimulated with PMA and ionomycin in the presence of Brefeldin A and Monensin for 2 hours and IFNγ production was quantified by intracellular cytokine staining. Bar chart depicts IFNγ+ CD8+ T cells. **(D)** CD8+ effector memory (Teff) cells were quantified by flow cytometry at day 8 by expression of CD44 and CD62L. **(E)** CD8+ central memory (TCM) cells were quantified by flow cytometry at day 16 by expression of CD44 and CD62L. Data is represented by mean ± SEM. P values were calculated by ordinary one-way ANOVA with the Tukey Kramer multiple comparison test, and P < 0.05 is considered statistically significant. * = < 0.05, ** = < 0.01,*** = < 0.001, **** = < 0.0001. NS, not significant. OVA, Ovalbumin; TLR, Toll-like receptor; PBMC, peripheral blood mononuclear cells.

Following our observation that AnnV-IL2 led to improved T cell activation *in vitro* and induced higher Ki67 expression *in vivo*, we questioned whether AnnV-IL2 *in vivo* administration would alter the CD8 T cell activation process. To better visualize and monitor expansion of antigen-specific cells, we utilized an adoptive transfer system with transgenic OT-1 CD8 T cells. We treated the mice with the peptide corresponding to the immunodominant epitope of ovalbumin (SIINFEKL) and a TLR9 agonist to drive OT-1 cell activation. Mice were then treated once daily for five days with AnnV-IL2 or IL-2 (**Figure 4A**). Peripheral blood was collected on day 8 and antigen-specific cells were enumerated by tetramer staining. AnnV-IL2 treated mice had approximately a 2-fold greater expansion of OVA-specific CD8 T cells and, consequently, a higher frequency of IFNγ+ cells (**Figures 4B, C**). Furthermore, AnnV-IL2 treated mice had higher expression of CD44+ T effector CD8 cells on day 8, and CD44+ CD62L+ central memory CD8 T cells on day 16 (**Figures 4D, E**). Consistent with our *in vitro* results, AnnV-IL2 treatment was able to increase the magnitude of the primary CD8 T cell response. Additionally, we monitored the weight of the mice for the duration of the experiment and observed no significant changes (**Supplementary Figure 2C**). Together, this data suggests that AnnV-IL2 is more effective than IL-2 at driving expansion of activated CD8+ T cells.

### AnnV-IL2 Treatment Supports Development of a Larger Antigen-Specific Memory Pool

**Figure 5:**
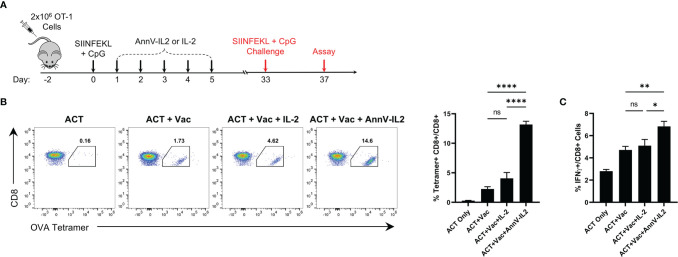
AnnV-IL2 promotes larger memory cell pool capable of enhanced secondary expansion. **(A)** Schematic of experimental design. Naive C57BL/6 mice received 2x106 OT-1 cells intravenously on day -2, followed by intraperitoneal injection of the peptide corresponding to the immunodominant epitope of OVA (OVA257-264; SIINFEKL) and TLR9 agonist, CpG, on day 0. Mice were treated with equimolar amounts of AnnV-IL2 or IL-2 intraperitoneally once daily for a total of 5 days. Mice were challenged with SIINFEKL peptide and CpG on day 33. N=5 mice per group. **(B)** PBMC’s were isolated on day 37 and antigen specific cells were quantified by tetramer staining. Dot plots are representative of one mouse in each group. Bar chart depicts levels of OVA257-264-specifc CD8+ T cells. **(C)** PBMC’s were stimulated with PMA and Ionomycin in the presence of Brefeldin A and monensin for 2 hours and IFNγ production was quantified by intracellular cytokine staining. Bar chart depicts IFNγ+ CD8+ T cells. Data is represented by mean ± SEM. P values were calculated by ordinary one-way ANOVA with the Tukey-Kramer multiple comparison test, and P < 0.05 is considered statistically significant. * = < 0.05, ** = < 0.01, **** = < 0.0001. NS, not significant. OVA, Ovalbumin; TLR, Toll-like receptor; PBMC, peripheral blood mononuclear cells.

Following the peak of the primary immune response, approximately 95% of antigen-specific T cells undergo programmed cell death and a small population of T cells emerge as long-lived memory cells poised to rapidly respond to a rechallenge (29, 30). Since AnnV-IL2 was able to increase the magnitude of the primary response, we inferred that there would be a higher frequency of antigen-specific memory cells following contraction (30). In return, AnnV-IL2 treated mice would be capable of a stronger recall response upon rechallenge. After the memory cell population was established, antigen experienced mice were rechallenged with an identical dose of SIINFEKL peptide plus CpG adjuvant (**Figure 5A**). Four days after the rechallenge, peripheral blood was collected, and the antigen specific recall response was quantified by tetramer staining. Mice who were treated with AnnV-IL2 during the primary response had a significantly higher frequency of OVA-specific CD8 T cells compared to mice who received IL-2 (**Figure 5B**). Additionally, AnnV-IL2 treated mice had a higher frequency of IFNγ+ CD8 T cells (**Figure 5C**). This data indicated that AnnV-IL2 treatment during the primary immune response leads to a larger memory cell population. Memory cells are critical for protecting against reinfection with a specific pathogen and for controlling tumor progression. AnnV-IL2 treatment during the early activation phase potentially provides superior long term immune protection compared to IL-2.

## Discussion

Our findings support the notion that T cell membrane lipid composition changes in response to TCR engagement and PS localizes to the external leaflet. We showed that the PS-AnnV axis can be leveraged to deliver protein payloads, such as IL-2, to aid T cell activation. Following *in vitro* activation, AnnV-IL2 anchored to PS on the surface of CD8 T cells and augmented expansion of activated T cells compared to IL-2. Our findings were paralleled *in vivo*, where we observed greater expansion of adoptively transferred OT-1 cells after peptide vaccination and treatment with AnnV-IL2. Of note, AnnV-IL2 treatment led to a larger memory cell population capable of greater expansion upon secondary exposure to antigen, which is an important aspect of long-term immunity (30). Our findings have relevant translational implications where T cell mediated immunity is critical, including vaccines and cancer immunotherapy.

Triggering of the IL-2 receptor results in the activation of several common signaling pathways that drive cell cycle progression, transcription of key T cell genes and protein synthesis (4). CD8 T cells isolated from AnnV-IL2 treated mice showed a significantly higher expression of Ki67, a nuclear protein that is upregulated during cell division (28). In that regard, tethering IL-2 to the T cell surface may affect the duration or magnitude of signaling through the IL-2R and contribute to the proliferation that we observe both *in vitro* and *in vivo*. Moreover, fusion of IL-2 to AnnV may alter the pharmacokinetic and biodistribution profile of IL-2 in the *in vivo* setting. Fluorescence imaging following *in vivo* administration revealed that AnnV-IL2 was able to accumulate and persist in the spleen where CD8 T cells reside and undergo activation by antigen presenting cells. In that regard, sustained IL-2R activity as a result of AnnV-IL2 in close proximity to CD8 T cells may contribute to the increased number of CD8 T cells we observed in mice. Although the biological properties of IL-2 remained unaltered, our construct is engineered to bias IL-2 towards activated T cells and provide heightened specificity *in vivo*. Additionally, one caveat of conventional IL-2 therapy is binding of IL-2 to off-target cells, such as endothelial cells, that express the IL-2R (31). Off-target IL-2 activity can result in vascular leak syndrome in patients receiving high-dose IL-2 therapy (32, 33). The enhanced immune responses observed after *in vivo* AnnV-IL2 treatment compared to IL-2 may be a result of direct delivery of IL-2 to CD8 T cells and reduced off-target consumption of IL-2. While our data does not provide direct evidence, future research into the pharmacokinetics and biodistribution of AnnV-IL2 will help elucidate the mechanism for how this molecule modulates CD8 T cell immunity. Interestingly, NK cell populations expanded *in vitro* following AnnV-IL2 or IL-2 treatment but remained stable following *in vivo* administration. NK cells express IL-2Rβγ and are known to proliferate in response to IL-2 (34). NK cells are innate immune cells that depend heavily on cytokines for development and cytotoxic activity and are an important bridge between the innate and adaptive immune systems (34, 35). The protein dose and timing of delivery are critical factors for *in vivo* efficacy, and we think the treatment schedule used in our studies may not be sufficient to observe peripheral NK cell expansion. Future studies will evaluate the effects of various doses of AnnV-IL2 on the NK cell compartment.

While our results support the use of AnnV-IL2 for targeting T cell activation pathways, IL-2 can be substituted with other cytokines that impact T cell functionality. IL-7 and IL-15 are additional cytokines in the y_c_ family and are important for regulating homeostatic proliferation as well as survival and effector function of CD8 memory T cells (36, 37). We determined that antigen experienced cells externalize PS in response to TCR stimulation faster and to a greater degree than antigen inexperienced CD8 T cells. Considering the central role of IL-7 and IL-15 in memory T cell biology, an AnnV-IL7/IL15 fusion could be employed to aid the transition from effector to memory phenotype or augment secondary immune responses upon exposure to an identical antigen. In contrast, IL-10 is important for immune regulation and has a role in suppressing T cells activated against self-antigens (38). In that regard, IL-10 can be combined with AnnV to target self-reactive CD8 T cells that are responsible for tissue destruction in autoimmune disease. One key feature of our AnnV-based chimeric fusion platform is that it can be precisely tailored to drive specific T cell phenotypes.

Due to the enhanced immune activation as a result of targeted IL-2 delivery to T cells, AnnV-IL2 has relevant translational implications in the context of anti-cancer therapies. CD8 T cells are critical for surveilling tissue to identify and eliminate malignant cells. For those reasons, the ability to incite a robust anti-tumor CD8 T cell response is a central focus of many cancer immunotherapeutic strategies. IL-2 has been well established as a T cell growth factor and has been widely implemented to support strong anti-tumor T cell responses (25). IL-2 has revolutionized adoptive cell therapy approaches and been used in combination with other cytokines, cell-based therapies, chemotherapy, peptide vaccines, checkpoint inhibitors and targeted therapies (39). Our findings suggest that AnnV-IL2 has a more attractive therapeutic profile compared to IL-2 and may demonstrate improved efficacy in combination with adoptive cell transfer or therapeutic vaccine strategies.

In addition to activated T cells, the tumor microenvironment (TME) has been shown to be a source of externalized PS. The competition for nutrients, hypoxia, acidity, calcium influx, and the presence of reactive oxygen species creates a stressful environment that results in exposed PS on endothelial cells, tumor cells, stromal cells, and tumor derived microvesicles (40–42). Moreover, apoptotic cell death in response to cytotoxicity, chemotherapy or radiation contributes to the density of PS in the TME (40–43). Binding of PS and PS receptors (PSR) has a number of consequences that permits an immunosuppressive environment (44–47). To that end, PS is considered an upstream immune checkpoint that contributes to tumor progression and targeting PS-PSR interactions with AnnV has been shown to increase efficacy of cancer immunotherapies (48). To that end, we infer that AnnV-IL2 would localize to tumors given that AnnV binds to PS. Consequently, AnnV-IL2 may interrupt PS-mediated immunosuppression in the TME. Moreover, delivery of IL-2 to the TME as a result of AnnV-mediated tumor localization could support survival and effector functions of tumor infiltrating lymphocytes. Future studies should investigate the anti-tumor properties of AnnV-IL2 and evaluate efficacy compared to conventional IL-2 therapy.

It is well understood that IL-2 is positioned at the interface of T cell activation and tolerance and has been developed for applications in both oncology and autoimmunity (49, 50). Tregs constitutively express high levels of IL-2Rα and are readily available to consume IL-2 in the environment. Although Tregs are imperative for protecting against overactivation of the immune system during infection, Tregs are detrimental in the context of anti-tumor immune responses and may impact the therapeutic application of AnnV-IL2 (51). We observed a brief spike in Treg frequency following delivery of AnnV-IL2 *in vivo* but this effect was short-lived while IL-2 treated mice continued to have elevated Treg populations. To bypass Treg activation in the presence of IL-2, biased agonists have been developed by eliminating binding of IL-2 to IL-2Rα (52). Here, IL-2 is biased towards naïve and memory T cells as well as NK cells that bear IL-2Rβγ at resting conditions. IL-2 mutants display enhanced anti-tumor effects due to preferential activation of CD8 T cells and NK cells, and decreased expansion of Tregs (53, 54). Since our AnnV-IL2 construct is genetically engineered, incorporation of key IL-2Rα amino acid mutations could circumvent Treg-related complications. Comparison of wild type and mutant AnnV-IL2 will be important for understanding how our construct impacts IL-2 responsive cell populations, and whether selective binding improves therapeutic efficacy. In addition to the role of IL-2 in supporting Treg development and function, IL-2 plays other important immunoregulatory roles. It has been shown that IL-2 signaling during T cell activation sensitizes the cell to activation induced cell death in the event of recurrent TCR stimulation as a mechanism to control for expansion of autoreactive T cells (55). This process has been shown to be mediated in part by IL-2 induced activation of the cytotoxic Fas/Fas Ligand pathway (56). Although our data suggests that AnnV-IL2 supports expansion of CD8 T cells following TCR activation, further studies will seek to uncover any immunoregulatory properties of AnnV-IL2.

While our results suggest that AnnV-IL2 can expand activated OT-1 cells following antigen receptor mediated activation, we acknowledge that our proof-of-principle system does not facilitate reporting on other immune cell populations because administration of SIINFEKL peptide limits our analysis to MHC Class I restricted T cells. There is reason to believe that CD4 T cells would be responsive to administration of AnnV-IL2 because conventional CD4 T cells express the IL-2R and have been shown to externalize PS in response to TCR stimulation (4, 19, 50). Model systems that rely on endogenous processing and presentation of antigen on both MHC class I and II, such as in viral or tumor models, will provide a deeper understanding of how AnnV-IL2 modulates immunity. Furthermore, use of viral or tumor models will provide an opportunity to better address how AnnV-IL2 administration impacts other correlates of immune responses such as various memory cell subsets, T cell exhaustion and T cell polyfunctionality. Future studies will validate the use of AnnV-IL2 in these model systems and lend further insight into the therapeutic efficacy of our recombinant protein.

In summary, our data supports the use of AnnV-based fusion proteins as a method to deliver protein payloads to PS+ T cells. We demonstrated that AnnV-IL2 was able to successfully modulate CD8 T cell expansion following TCR activation. Due to this property, AnnV-IL2 has potential utility in cancer immunotherapeutic strategies and may be a feasible alternative to traditional IL-2 therapy.

## Materials and Methods

### Mice

Six-week-old female C57Bl/6 and OT-1 transgenic mice were purchased from Taconic biosciences (Cambridge City, IN). All mice were housed in sterile conditions at the Johns Hopkins University School of Medicine Oncology Animal Facility in Koch Cancer Research Building II (Baltimore, MD). All animal studies adhered to approved protocols from the Johns Hopkins Institutional Animal Care and Use Committee and were in accordance with recommendations for proper use and care of laboratory animals.

### Cell Culture

Antigen experienced memory cells were generated by repeatedly culturing OT-1 cells with irradiated TC-1 tumor cells pulsed with OVA_257-264_ peptide. Antigen inexperienced and experienced cells were grown *in vitro* in complete RPMI-1640 media (RPMI-1640 supplemented with 10% fetal bovine serum, 50 units/mL of penicillin/streptomycin, 2 mM of L-glutamine, 1 mM of sodium pyruvate, and 2 nM of non-essential amino acids) (Gibco, Waltham, MA) and grown at 37°C and 5% CO2. CTLL2 cells were purchased from ATCC (Manassas, VA) and were grown *in vitro* in complete RPMI media supplemented with IL-2 according to established protocols (57).

### PS Exposure on T Cells

1x10^5^ antigen inexperienced cells were cultured in a 96 well U-bottom plate (Genesee Scientific, San Diego, CA) with varying concentration of plate bound anti-CD3 (0.25, 0.5, 1.0, 2.0 μg/mL) (Clone 2C11, BioXCell, Lebanon, NH) and 1.0 μg/mL soluble anti-CD28 (Hamster-anti-mus Clone 37.51, BioXCell, Lebanon, NH). 1x10^5^ antigen experienced cells were cultured with 1.0μg/mL anti-CD3/anti-CD28. Cells were collected at 0, 6, 12, 24, and 48 hours (0, 6, 24, 48 hours for antigen experienced) and stained with AnnexinV-Alexa647, CD69-PE, CD8-BV785 prepared in 1x Annexin-V binding buffer (BD Biosciences, Franklin Lakes, NJ) for 30 minutes at 4°C. Cells were washed and resuspended in 1x Annexin V binding buffer containing 7-AAD viability dye (Biolegend, San Diego, CA) and acquired on a CytoFLEX S flow cytometer (Beckman Coulter, Brea, CA).

### Annexin V Sorting

Primary CD8 T cells were isolated from naïve OT-1 mice (described below) and cells enumerated by a Countess II FL cell counter (Invitrogen, Waltham, MA). Cells were cultured for 48 hours with 3.0μg/mL plate bound anti-CD3. PS exposure was confirmed by staining AnnV-PE, CD69-APC and CD8-BV421 before proceeding. Cells were pooled together and stained with AnnV-PE and AnnV+ cells were isolated by positive selection (Release PE Positive Selection Kit, Stem Cell Technologies, Cambridge, MA) following manufactures protocol.

### EC50 – pSTAT5

CTLL2 cells were collected, washed with fresh complete RPMI-1640 media and cultured with complete RPMI-1640 media free of IL-2 for 5 hours. After 5 hours, 1x10^5^ CTLL2 cells were stained for 10 minutes with Zombie Aqua Fixable Viability stain (Biolegend, San Diego, CA). Cells were then stimulated for 15 minutes with AnnV-IL2 or IL-2 prepared in complete RPMI media using a 3-fold serial dilution. After 15 minutes, 1:1 volume of 8% PFA in PBS/3% MeOH was immediately added to each well and incubated at RT for 10 minutes. Cells were washed with MACS buffer (0.05% BSA in PBS) and resuspend with ice cold Perm III buffer (BD Biosciences, Franklin Lakes, NJ) and incubated overnight at -20°C. 5ul from each sample was collected for the isotype control. Cells were washed 2x with MACS buffer and stained with pSTAT5-Alexa647 for 30 minutes at 4°C. Cells were washed with MACS and samples acquired on a CytoFLEX S flow cytometer.

### DNA Construct, Protein Expression, and Purification

To generate pET28-IL2, mouse IL2 was first amplified by PCR using pcDNA3-IL2 described previously (58) as a template and the following set of primers: 5’- TTTGAATTCGCACCCACTTCAAGCTCCAC -3’ and 5’- TTTCTCGAGTTGAGGGCTTGTTGAGATGA -3’. The PCR product was cloned into the EcoRI and XhoI sites of the pET28a vector. To generate pET28- AnnV-IL2, the PCR product was cloned into EcoRI and XhoI sites of pET28- AnnV described previously (48). The DNA plasmids were confirmed by sequencing and transformed into Escherichia coli [BL21(DE3)] for protein expression. The recombinant protein was purified by Ni+ affinity chromatography (Ni-NTA agarose, Qiagen) according to the methods described previously (48).

### Generation of Fluorescent AnnV-IL2 and IL-2

AnnV-IL2 or IL-2 proteins were labeled with Alexa-647 dye according to the manufacturers instructions (Invitrogen, Waltham, MA). Free dye was removed using a 7,000 Da MW Zebra Spin Desalting Column (Thermo Fisher Scientific, Waltham, MA).

### Phosphatidylserine-AnnV-IL2 Binding

1.5x10^5^ OT1 *in vitro* generated memory cells were stimulated for 5 hours with 1.0μg/mL plate bound anti-CD3 and soluble anti-CD28 antibodies. After 5 hours, cells were stained for 10 minutes with Zombie Aqua Viability Dye. Cells were resuspended in 1x Annexin V binding buffer or Annexin V binding buffer containing 50μg/well of unlabelled AnnV protein. Cells were incubated for 30 minutes at room temp and then washed with 1x Annexin V binding buffer. Cells were stained with Alexa-647-AnnV-IL2 protein prepared in Annexin V binding buffer for 30 minutes. Cells were washed with Annexin V binding buffer and samples acquired on a CytoFLEX S flow cytometer.

### *In Vitro* CD8 Characterization

For cell proliferation analysis, 1-2x10^5^ primary cells or *in vitro* generated memory cells were stimulated with 0.1-0.25μg/mL plate bound anti-CD3 and 0.1-.25μg/mL soluble anti-CD28 for 24 hours. After 24 hours, media was removed and replaced with media supplemented with equimolar amounts of Annv-IL2 (3-9ng/mL) or IL-2 (1-3ng/mL) and 0.25μg/mL anti-CD28 (primary cells). Cells incubated for an additional 4-6 days at 37°C. After 4 days, primary cells were harvested and stained with 7-AAD viability dye. *In vitro* generated memory cells were supplemented with media containing AnnV-IL2 or IL-2 on day 4 and collected on day 6. Cells were washed and resuspended in MACS buffer and 20μL of sample was collected on a CytoFLEX S Flow Cytometer for counting.

For CD8 T cell effector function analysis, isolated primary CD8 T cells (see below) were cultured in a 96 well U-bottom plate with plate bound anti-CD3 (0.1μg/mL) and soluble anti-CD28 (0.1μg/mL) for 3 days at 37°C. Media was supplemented with 150ng/mL AnnV-IL2 protein or 50ng/mL IL-2 for respective groups. Antigen experienced cells were cultured with plate bound anti-CD3 (0.1μg/mL) or SIINFEKL peptide (10pg/mL-2ug/mL) for 24 hours in media supplemented with 150ng/mL AnnV-IL2 protein or 50ng/mL IL-2. Brefeldin A (Invitrogen, Waltham, MA) was added to culture and incubated for 6 hours prior to downstream flow cytometry analysis. Cells were stained with Zombie aqua viability dye, CD8-BV421, and IFNγ-APC.

Cell culture supernatant was collected and stored at -20°C. Supernatant was diluted with fresh RPMI-1640 media before use. IFNγ production was quantified by mouse IFNγ ELISA (BD Biosciences, Franklin Lakes, NJ), kit following manufactures instructions.

### Primary CD8 T Cell Isolation

Spleens and lymph nodes were removed from naïve OT-1 or C57Bl/6 mice into complete RPMI media. Single cell suspensions were generated by dissociation of tissue with a syringe plunger (Fisher Scientific, Waltham, MA) and passed through a 70μm filter. Cells were centrifuged at 500xg and resuspended in PBS containing 2% FBS and 1mM EDTA, and CD8 T cells were negatively selected using a mouse naïve CD8 T cell isolation kit (Stem Cell Technologies, Cambridge, MA) following manufactures protocol.

### Adoptive Transfer and Rechallenge

Naïve OT-1 CD8 T cells were isolated as described above and washed extensively with sterile PBS before use. 2x10^6^ OT-1 cells prepared in 100μL of sterile PBS were injected intravenously by retroorbital injection into naïve C57Bl/6 mice on day -2. Mice were treated intraperitoneally with 10μg of peptide corresponding to the immunodominant epitope of ovalbumin protein (SIINFEKL, OVA_257-264)_ (GenScript, Piscataway, NJ) and 30μg of the TLR9 agonist CpG (GenScript, Piscataway, NJ) prepared in sterile PBS on day 0. Mice were intraperitoneally injected with equimolar amounts of AnnV-IL2 or IL-2 protein prepared in sterile PBS on days 1-5. On day 33 following the initial challenge with SIINFEKL peptide and CpG, mice were challenged intraperitoneally with an identical dose of SIINFEKL peptide and CpG.

### Preparation of PBMCs for Flow Cytometry Analysis

For peripheral immune profiling, blood samples were collected by submandibular bleeding into Eppendorf tubes containing 10% EDTA. Red blood cells were then lysed twice with RBC Lysis Buffer (Santa Cruz Biotechnology, Dallas, TX) and washed with MACS buffer. Lymphocytes were filtered with a 70μM filter before use for downstream analysis. For intracellular cytokine quantification, cells were stimulated with PMA/Ionomycin cell stimulation cocktail (Invitrogen, Waltham, MA) in the presence of Brefeldin A (Biolegend, San Diego, CA) and Monensin (Biolegend, San Diego, CA) for 2 hours at 37°C prior to downstream analysis.

### Flow Cytometry

All samples were stained with Zombie Aqua Fixable Viability Dye unless otherwise indicated. Cells were Fc blocked prior to extracellular staining (BioXcell, Lebanon, NH). For intracellular staining, cells were fixed and permeabilized with the FoxP3/Transcription factor staining buffer set (Invitrogen, Waltham, MA) according to manufactures instructions prior to staining with antibodies prepared in FoxP3 buffer. All samples were acquired on a CytoFLEX S machine. All antibodies used in our analysis can be found in **Table 1**. Compensation was generated using single-staining controls and CytExpert 2.0 software (Beckman Coulter, Brea, CA). Negative gates were determined using appropriate unstimulated controls or isotype controls. Analysis was performed using FlowJo v10 (BD Biosciences, Franklin Lakes, NJ).

**Table 1:** Flow antibodies.

Antibody	Catalog #	Manufacturer
pSTAT5-Alexa-647 (pY694)	612599	BD Biosciences
OVA Tetramer-PE		MBL
CD44-PE-Dazzle	103056	Biolegend
CD62L-APC-R700	565159	BD biosciences
CD8-BV421	100737	Biolegend
CD4-BV785	100552	Biolegend
IFNg-FITC	100706	Biolegend
CD25-PE-Cy7	101916	Biolegend
FoxP3-APC	126408	Biolegend
NK1.1-APC-Fire750	108752	Biolegend
Ki67-BV421	652411	Biolegend
CD8-BV650	100742	Biolegned
AnnV-PE	640908	Biolegend
CD69-APC	104514	Biolegend
IFNg-APC	505810	Biolegend

### *In Vivo* Fluorescence Imaging

AnnV-IL2 or IL-2 protein were labeled with Alexa-647 dye according to manufactures instructions (Thermo Fisher Scientific, Waltham, MA). Equimolar amounts of protein were injected intravenously through the retroorbital vein. After 4 hours, mice were euthanized and the spleen, lymph nodes, liver and kidneys were removed and immediately imaged *via* the *via* the IVIS^®^ Spectrum *in vivo* imaging system series 2000 (PerkinElmer).

### Statistical Analysis

All statistical analysis was performed using GraphPad Prism V9 (San Diego, CA). All data is reported as mean ± SEM. For all data displayed in a bar graph, results were analyzed using a one-way analysis of variance (ANOVA) test and the Tukey-Kramer multiple comparison test. P < 0.05 is considered statistically significant. Note: * = < 0.05, ** = < 0.01,*** = < 0.001, **** = < 0.0001.

## Data Availability Statement

The raw data supporting the conclusions of this article will be made available by the authors, without undue reservation.

## Ethics Statement

The animal study was reviewed and approved by Johns Hopkins Institutional Animal Care and Use Committee.

## Author Contributions

Conception and design: AM, CFH. Conducting experiments: AM, BL, JL, YJK, YCT. Analysis and interpretation of data: AM, BL TCW. Writing and review of manuscript: AM, BL, CFH, LF. Study supervision: CFH. All authors contributed to the article and approved the submitted version.

## Funding

This study was supported by the National Institutes of Health, National Cancer Institute Specialized Program of Research Excellence (SPORE) in Cervical Cancer grant (NIH/NCI P50CA098252). In addition, this work was supported by NIH/NCI R01CA233486 and pilot-projects R21CA234516 and R21DE029910. Brandon Lam is a recipient of an NIH-supported career development fellowship (5F31CA236051).

## Conflict of Interest

T-CW is a co-founder of and has an equity ownership interest in Papivax LLC. Also, T-CW owns Papivax Biotech Inc. stock options and is a member of Papivax Biotech Inc.'s Scientific Advisory Board.

The remaining authors declare that the research was conducted in the absence of any commercial or financial relationships that could be construed as a potential conflict of interest.

## Publisher’s Note

All claims expressed in this article are solely those of the authors and do not necessarily represent those of their affiliated organizations, or those of the publisher, the editors and the reviewers. Any product that may be evaluated in this article, or claim that may be made by its manufacturer, is not guaranteed or endorsed by the publisher.
