# Supplementary material for: Delivery of IL-2 to the T Cell Surface Through Phosphatidylserine Permits Robust Expansion of CD8 T Cells
Source: Front Immunol. 2021 Nov 4;12:755995. doi: 10.3389/fimmu.2021.755995 (PMC8599986; doi:10.3389/fimmu.2021.755995)
Supplement: Supplementary file 1 [file DataSheet_1.pdf]

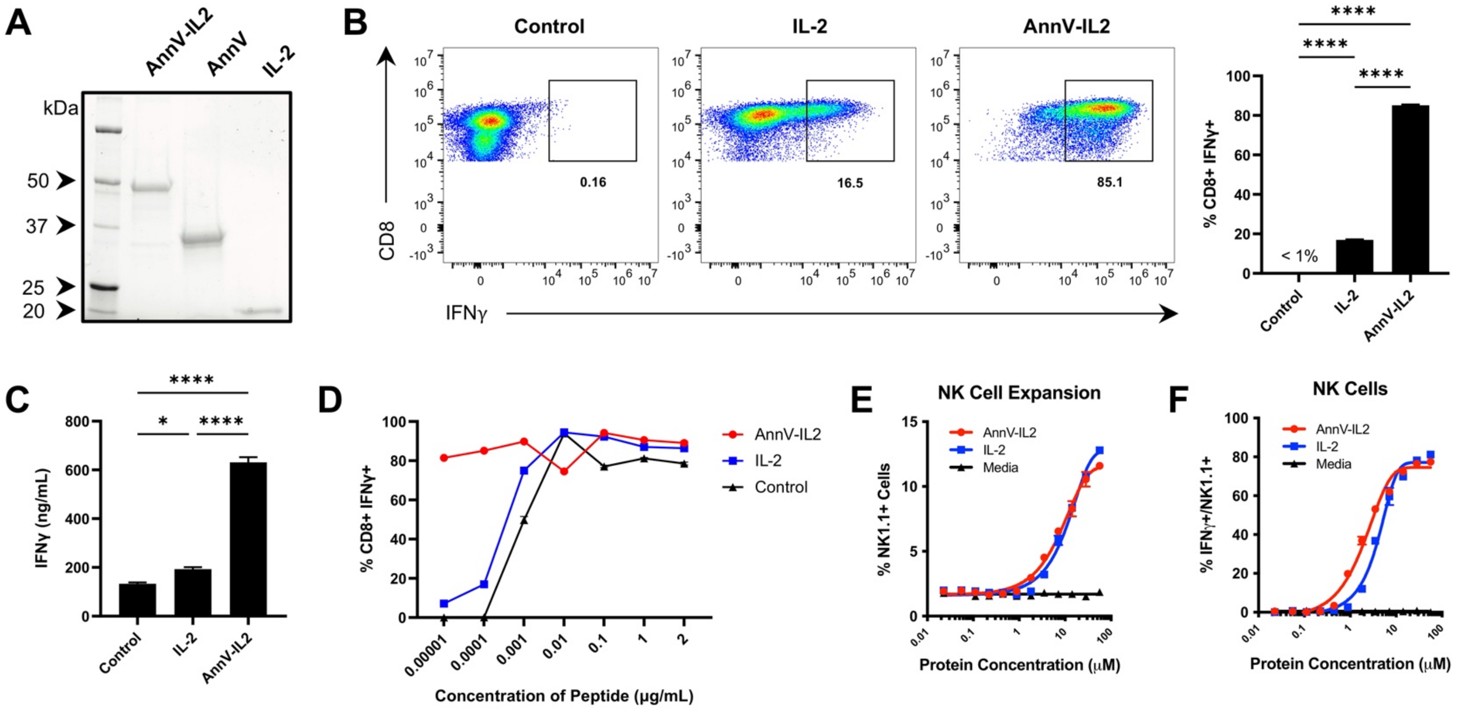

Supplement Fig. 1: A) Protein gel showing molecular weight of AnnV-IL2, AnnV and IL-2 proteins. B-C) Antigen experienced OT-1 T cells were cultured with SIINFEKL peptide in the presence of equimolar amounts of AnnV-IL2 or IL-2 for 24 hours. Brefeldin A was added to culture media in the final 6 hours of culture. B) Representative flow plot and quantification of IFNγ at 100pg/mL of SIINFEKL by intracellular flow cytometry. C) ELISA for IFNγ in supernatant of OT- 1 cell culture. D) Quantification of IFNγ by intracellular flow cytometry at the indicated concentrations of peptide stimulation. E) Murine splenocytes were cultured with media, AnnV-IL2, or IL-2 supplemented media for 3 days and percentage of NK cells was determined by flow cytometry F) IFNγ production in NK cells at day 3 was quantified by intracellular flow cytometry. Data is represented by mean ± SEM. P values were calculated by ordinary one-way ANOVA with the Tukey-Kramer multiple comparison test, and P < 0.05 is considered statistically significant. * = < 0.05, **** = < 0.0001.

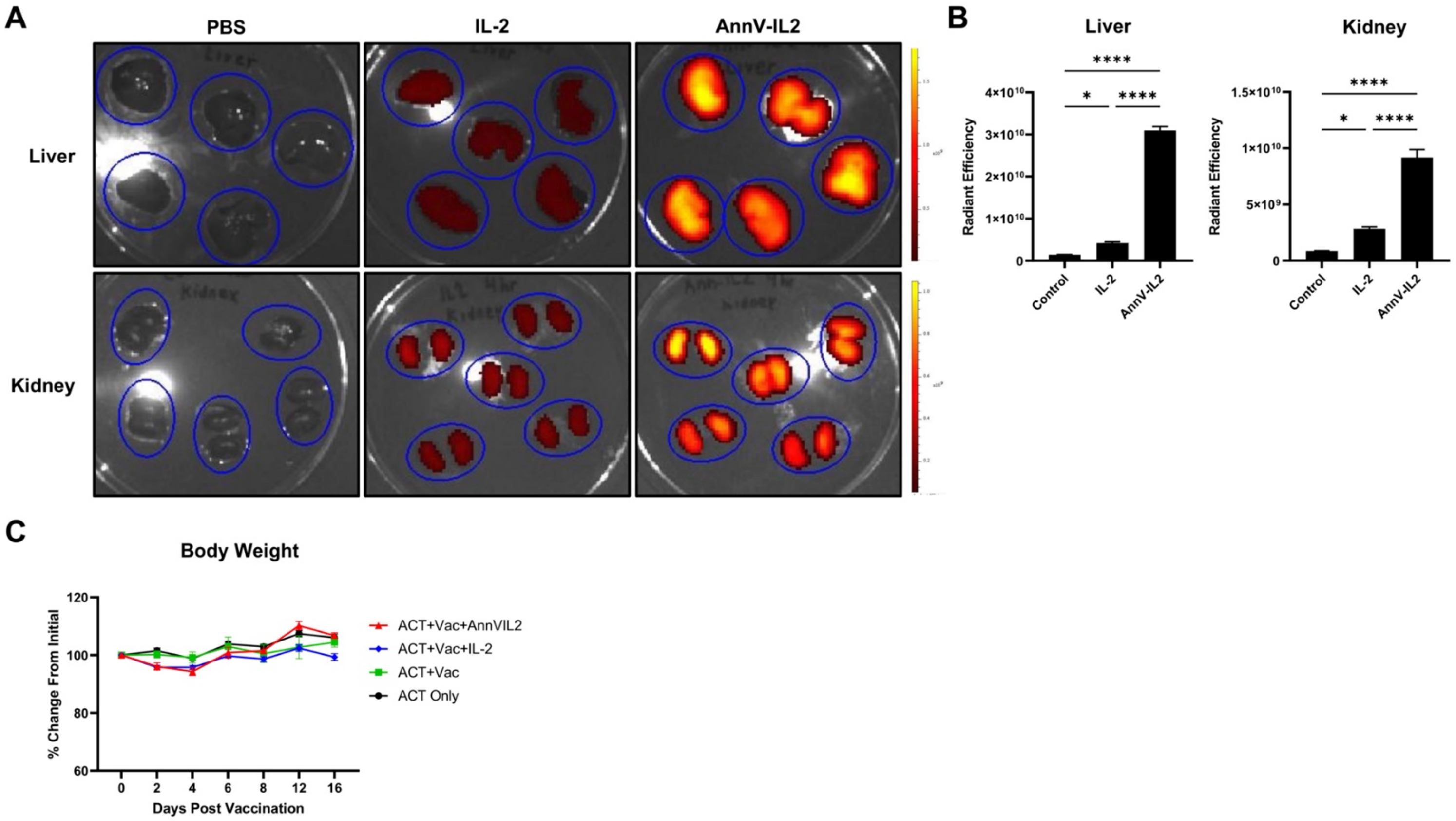

Supplement Fig. 2: A) Equimolar amounts of Alexa-647 labeled AnnV-IL2 or IL-2 protein was injected intravenously. After 4 hours kidney and liver were removed and imaged via IVIS imaging. Tissue is outlined in blue ovals. B) Quantification of fluorescence signal in each tissue. C) Naïve C57BL/6 mice received 2x106 OT-1 cells intravenously on day -2, followed by intraperitoneal injection of the peptide corresponding to the immunodominant epitope of OVA (OVA257-264, SIINFEKL) and TLR9 agonist, CpG, on day 0. Mice were treated with equimolar amounts of AnnV-IL2 or IL-2 intraperitoneally once daily for a total of 5 days. Weight of the mice was recorded on the indicated days. Data is represented by mean ± SEM. P values were calculated by ordinary one-way ANOVA with the Tukey-Kramer multiple comparison test, and $P < 0.05$ is considered statistically significant. * = < 0.05, **** = < 0.0001.
